# Substance Use among youths in Uganda during the COVID-19 pandemic: Associated factors and prevalence:

**DOI:** 10.1192/j.eurpsy.2023.656

**Published:** 2023-07-19

**Authors:** L. Ssenyonjo, I. Ddumba

**Affiliations:** ^1^ Research and Innovation, African Research center 4 Ageing & dementia; ^2^Nursing, Victoria University, Kampala, Uganda

## Abstract

**Introduction:**

Although studies have demonstrated that younger people are at high-risk of instigating alcohol, substance use and development of related disorders, the trend is gradually changing. An observed gradual initiation and substance use among older persons, especially during the COVID-19 pandemic. However, there is dearth of data on about the prevalence and determinant of substance use during COVID-19 pandemic in Uganda.

**Objectives:**

Determine the prevelence and determinants of susbstance use during COVID-19 pandemic in Uganda

**Methods:**

A cross-sectional design and probability based sampling was applied. Data was collected among 474 older persons aged 50 years and above, that resided in the central region of Uganda. A multivariate logistic regression was used to assess the socio-economic, demographic and health related correlates of alcohol and tobacco use.

**Results:**

9.2% and 5.4% of older persons were taking alcohol and smoking before the COVID-19 pandemic. However, an observed increase of 14% of smoking and alcohol intake among older persons during the pandemic. Being male had higher odds of substance use than their female counterparts. Older persons with tertiary education and low (poor) wealth quantile, had lower odds of substance use than their counterparts.

**Conclusions:**

Our finding highlights increased substance use among older persons during the COVID-19 pandemic. Designing targeted measures and policies to deter the substances use among older persons is critical to address this vice, especially during the pandemic and possible future disease outbreaks.

**Disclosure of Interest:**

None Declared

